# Optimizing the Long-Term Operating Plan of Railway Marshalling Station for Capacity Utilization Analysis

**DOI:** 10.1155/2014/251315

**Published:** 2014-11-26

**Authors:** Wenliang Zhou, Xia Yang, Jin Qin, Lianbo Deng

**Affiliations:** ^1^School of Traffic and Transportation Engineering, Central South University, Changsha 410075, China; ^2^Department of Civil and Environmental Engineering, Rensselaer Polytechnic Institute, Troy, AL 12180, USA

## Abstract

Not only is the operating plan the basis of organizing marshalling station's operation, but it is also used to analyze in detail the capacity utilization of each facility in marshalling station. In this paper, a long-term operating plan is optimized mainly for capacity utilization analysis. Firstly, a model is developed to minimize railcars' average staying time with the constraints of minimum time intervals, marshalling track capacity, and so forth. Secondly, an algorithm is designed to solve this model based on genetic algorithm (GA) and simulation method. It divides the plan of whole planning horizon into many subplans, and optimizes them with GA one by one in order to obtain a satisfactory plan with less computing time. Finally, some numeric examples are constructed to analyze (1) the convergence of the algorithm, (2) the effect of some algorithm parameters, and (3) the influence of arrival train flow on the algorithm.

## 1. Introduction

**Figure 1:**
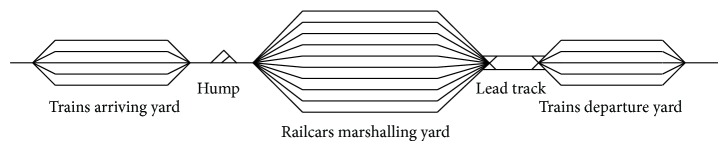
The layout of a typical marshalling station.

Railway marshalling station is the main place for disassembling and assembling trains in railway freight transport networks. Generally it can be divided into train arriving yard, railcar marshalling yard, and train departure yard consisting of many parallel tracks for different uses separately. The train arriving yard connects with the railcar marshalling yard by humps which are used to disassemble trains with gravitational pull, while railcar marshalling yard is connected to train departure yard by some lead tracks which allow for repeatedly assembling railcars. A typical marshalling station layout is shown in Figure 1, and the main operations can be described as follows.

1. Inbound trains enter the arriving yard and wait for disassembling.
2. Disassembling engine pushes inbound train through the hump after necessary technical inspections, and then the railcars from dissembling run on different marshalling tracks.
3. Assembling engines pull strings of railcars from marshalling tracks to the departure track to make up outbound trains.
4. Outbound trains depart from the departure yard after necessary technical inspections.

The above operations are entirely carried out according to a predetermined operating plan. It arranges the arrival track, the disassembling starting and ending time, the disassembling engine, and track assignments for each inbound train and the starting time, ending time, and the engine of assembling, the departure time, the component railcars, and storage track for each outbound train. The improvement of operating plan greatly contributes to decreasing railcars' staying time in station and enhancing station's operating performance. Besides, it has another important purpose of comprehensively analyzing the capacity utilization of a marshalling station, which is very beneficial for a railway company as it helps understand the station's limitations. According to a long-term operating plan, the general changing relationship between capacity utilization of each facility and some characteristics of arrival trains (e.g., arrival time distribution) can be obtained by repeatedly optimizing the long-term operating plan with different arrival train flow, which plays a significant role in the capacity-related decision making for a railroad company.

Generally, the operating plan of one day is divided into multiple time periods' plan, called stage operating plan, which arrange the inbound trains' disassembling, outbound trains' assembling, and shunting locomotive work. So far there are abundant studies on the stage operating plan. Li et al. [1] comprehensively reviewed the relative research on stage operating plans at marshalling stations; Gulbrodsen [2] was one among the first who studied the optimization of stage operating plan; Yagar et al. [3] studied the disintegration sequences of all arrival trains during all stages; Assad [4] considered the mutual interaction between different marshalling stations on the freight rail transportation network and presented work on train integration plan; Cicerone et al. [5] mainly worked on the planning of schedules during all stages; Shafia et al. [6] studied the robust of formation method for marshalling plans. In addition, some researchers, such as Hein [7], Petersen [8, 9], Turnquist and Daskin [10], and Dimitri [11], also further studied the operations, dwelling times, and delays at marshalling stations.

Compared with the abundant studies on stage plan optimization, there are much fewer studies on long-term operating planning. They are different in planning scale and marshalling purpose. The stage plan usually uses 3 hours as a stage, which is relatively small in scale and aims at providing reference for disassembling, assembling, and shunting locomotives. On the other hand, the long-term marshalling plan is mainly used in analyzing the equipment utilization conditions of hump, arrival yard, marshalling yard, and locomotives under various arrival train flows in order to discover the capacity inefficiency at the marshalling station in time. It covers a variety of arrival train flow densities and its scale is at least ten times even more as big as the stage plan.

However, achieving a fine operating plan is challenging as it covers too many interrelated decisions. It is NP-complete (see [12]). Most of researches struggled to obtain a better stage operating plan for guiding stations' operations, and the main methods they used include simulation optimization and heuristics search.

Simulation optimization is a typical method used to solve the operating plan problem. Gulbrodsen [2] firstly used it to optimize the stage operating plan. Lentink et al. [13] established a mathematical model of stage operating plan with network flow method. However, the simulation optimization method has a low efficiency in solving the operating plan problem due to the large scale.

Heuristics search algorithms have been applied in many fields nowadays as they can obtain a satisfactory solution with shorter computing time, although they also have difficulty in achieving the best solution. Shen et al. [14] designed an adaptive colonial selection algorithm out of the immune algorithm to solve the operating plan of railway marshalling yard. Li et al. [15] used the hybrid heuristic algorithm based on the harmony search strategy to optimize the stage operating plan.

Besides, Hein [7] and Turnquist and Daskin [10] applied the queuing theory to research the operating plan of marshalling yard and railcars staying time and their delays in marshalling yard. Ma et al. [16] designed a self-learning algorithm for conflict detection and adjustment to increase operating plan's fulfillment rates.

**Figure 2:**
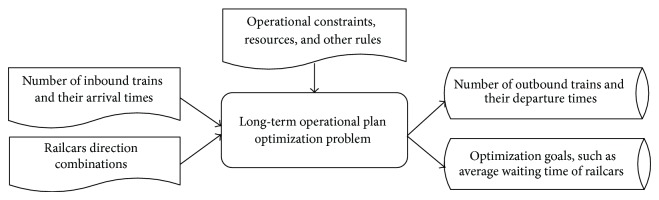
Long-term operating plan optimization problem.

Compared to the research of stage operating plan, very few researchers strived to optimize the long-term operating plan for analyzing the capacity utilization of marshalling station. In fact, it can provide time-varying details of capacity utilization while other analysis methods generally support a single utilization rate and so forth. This paper studies the optimization problem of long-term operating plan, as shown in Figure 2, and its main contributions are as follows. An optimization model of long-term operating plan is built firstly, and an efficient solving method is designed based on heuristics search (namely, GA) and simulation optimization. To be specific, in order to reduce the computing time, the long-term operating plan is divided into many subplans and then optimizes them one by one through combining subperiod rolling into GA.

This paper is organized as follows. The operating plan optimization problem formulation is firstly built in Section 2, and then its solution framework is given in Section 3. Under this framework, a simulation method of operating plan with a given assembling sequence of railcars is proposed in Section 4, and the optimization method combining subperiod rolling into GA is designed in Section 5. After that, the numerical analysis is provided in Section 6, and at last some conclusions are presented in Section 7.

## 2. Formulation of Operating Plan Optimization Problem

### 2.1. Notations and Assumptions

All symbols for optimizing operating plan are denoted as shown in Notations section.

All assumptions are given as follows.

**Assumption 1 .**

Each arrival track (departure track) only stores one inbound train (outbound train) at the same time and its length is enough to hold all railcars of any train.

**Assumption 2 .**

All railcars' size and shape are the same, and they are all allowed to be pushed to the hump for disassembling.

### 2.2. Optimization Goal

Minimizing railcars' total staying time from their arrival to departure at the station is the primary goal of operating plan optimization, which has been the focus in many studies on operating plans, for example, Lin and Cheng [17, 18]. Once railcars arrive at station with an inbound train, if they will stay in station until the end of plan horizon, railcars' total staying time is from their arrival time to the end of plan horizon; namely,

(1) $$\begin{array}{c} z_{l}=\overset{N}{\underset{i=1}{\sum }}\left(T-a_{i}\right)\left|B_{i}\right|. \end{array}$$

In fact, most railcars will assemble into an outbound train and depart from station at the end of plan horizon. Only a small part will stay in station. So the time from railcars departing from station to the end of plan horizon should be subtracted from *z*_*l*_, and railcars' actual staying time can be calculated as follows:

(2) $$\begin{array}{c} z=z_{l}-\overset{M}{\underset{j=1}{\sum }}\left[\left(T-d_{j}\right)\left|B_{j}\right|\right]. \end{array}$$

### 2.3. Constraints

#### 2.3.1. Used Track Number Constraints

At any time, train number in the arrival yard cannot exceed the total number of arrival tracks *U*, the number of trains in the departure yard should not be higher than the total number of departure tracks *W*, and the maximum number of used marshalling tracks is the capacity *V*:

(3) $$\begin{array}{c} \overset{U}{\underset{u=1}{\sum }}L_{u}\leq U,\\ \overset{W}{\underset{w=1}{\sum }}L_{w}\leq W,\\ \overset{V}{\underset{v=1}{\sum }}L_{v}\leq V, \end{array}$$

where *L*_*u*_, *L*_*w*_, *L*_*v*_ are the occupation signs of arrival track *u*, marshalling track *w*, and departure track *v*, respectively. If *i*_*u*_ > 0, then *L*_*u*_ = 1; otherwise *L*_*u*_ = 0. If *B*_*w*_ ≠ *∅*, then *L*_*w*_ = 1; otherwise *L*_*w*_ = 0. If *j*_*v*_ > 0, then *L*_*v*_ = 1; otherwise *L*_*v*_ = 0.

#### 2.3.2. Marshalling Track Capacity Constraint

The number of railcars on each marshalling track shall not exceed its storage capacity *ξ*; namely,

(4) $$\begin{array}{c} \left|B_{w}\right|\leq \xi ,\;w=1,2,\ldots ,W. \end{array}$$

And the number of railcars of inbound (outbound) train meets the arrival (departure) track capacity requirement according to Assumption 1.

#### 2.3.3. Task Order Constraint of Inbound Train

Inbound trains firstly enter into arrival yard for technical inspections and then are pushed up the hump for disassembling by engine. So inbound trains' arrival time, starting time, and ending time of disassembling must meet the following constraints:

(5) $$\begin{array}{c} a_{i}\leq e_{i},\;i=1,2,\ldots ,N,\\ e_{i}+\tau \leq h_{i}^{s},\;i=1,2,\ldots ,N,\\ h_{i}^{s}+\frac{\left|B_{i}\right|}{\delta }=h_{i}^{e},\;i=1,2,\ldots ,N. \end{array}$$

#### 2.3.4. Task Order Constraint of Outbound Train

All outbound trains stay in the departure yard for technical inspections after being assembled by combining some strings of railcars from marshalling yard and then depart from there. Therefore outbound trains' starting time and ending time of assembling and departing time must meet the following constraints:

(6) $$\begin{array}{c} \max\left\{h_{i_{b}}^{e}\;|\;b\in B_{j}\right\}\leq r_{j}^{s},\;j=1,2,\ldots ,M,\\ r_{j}^{s}+\epsilon_{f}+\left(n_{j}-1\right)\cdot \epsilon_{a}=r_{j}^{e},\;j=1,2,\ldots ,M,\\ r_{j}^{e}+\tau \leq d_{j},\;j=1,2,\ldots ,M. \end{array}$$

For simplicity, railcars strings' pull time described here only distinguishes first track pull and additional track pulls, regardless of the number of railcars of each track pull.

#### 2.3.5. Minimum Time Interval Constraints

Any two same type tasks including disassembling, assembling, and trains' departure must meet corresponding minimum time interval requirements; namely,

(7) $$\begin{array}{c} h_{i^{\prime }}^{s}-h_{i}^{e}\geq HI,\;\forall i^{\prime }\neq i,\\ r_{j^{\prime }}^{s}-r_{j}^{e}\geq AI,\;\forall j^{\prime }\neq j,\\ d_{j+1}-d_{j}\leq DI,\;j=1,2,\ldots ,M-1. \end{array}$$

#### 2.3.6. Outbound Train Size and Railcar Direction Combination Constraints

The outbound railcars direction combination specifies which railcars can be put together and their order on a departure train. For example, the feasible direction combination “A1, A2” means that outbound trains can be formed with railcars “A1” or “A2” or “A1, A2.” Therefore, all railcars constituting outbound train *j* must belong to a given direction combination; namely,

(8) $$\begin{array}{c} d_{b}\in c\in C,\;\forall b\in B_{j}. \end{array}$$

Meanwhile, the number of railcars of each outbound train must meet the minimum and maximum requirements; namely,

(9) $$\begin{array}{c} \Gamma_{\min}\leq \left|B_{j}\right|\leq \Gamma_{\max},\;j=1,2,\ldots ,M. \end{array}$$

#### 2.3.7. Railcars to Track Assignment Constraints

One marshalling track can be only assigned to railcars of one direction at any time. Railcars of any other direction are allowed to stay in the marshalling track after it is cleared:

(10) $$\begin{array}{c} d_{b}=d_{b^{\prime }},\;\forall b,b^{\prime }\in B_{w};\;\;w=1,2,\ldots ,W. \end{array}$$

## 3. Solution Framework Based on GA and Simulation

In order to minimize railcars' staying time in station, inbound trains should be disassembled immediately once they enter the arrival yard, and railcars should be assembled into new outbound trains once they meet all assembling requirements. In fact, some inbound trains cannot be disassembled in time because of the capacity limitation of disassembling engine. Likewise, some railcars cannot be assembled into outbound trains timely due to the capacity limitation of assembling engine. So the following two problems have to be solved in the first place.

1. Which inbound train should be disassembled first, which is equal to determining the disassembling sequence of inbound trains?
2. Which railcars should be first assembled into an outbound train, which means to confirm the assembling sequence of railcars?

**Figure 3:**
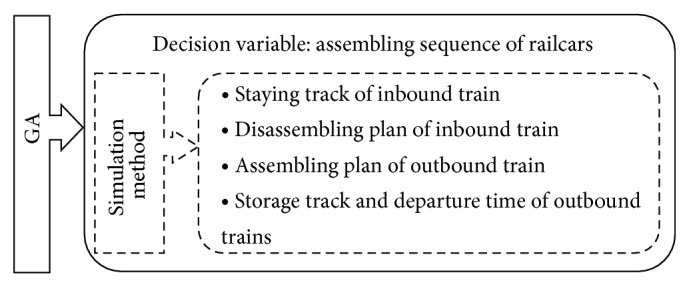
The solution framework of operating plan.

If the disassembling sequence of inbound trains is predetermined, it is a priority to assemble railcars whose directions belong to the same combination and maximize the number of railcars to an outbound train. Similarly, if the assembling sequence of railcars is pregiven, it should firstly disassemble inbound train with the maximum railcars assembled into the next outbound train. Compared with railcars' assembling sequence, it is more difficult to optimize the disassembling sequence of inbound trains as it has more relative constraints, such as inbound trains' arriving sequence and arrival track number. Thus, GA is chosen to optimize railcars' assembling sequence in the paper, and, based on each acquired assembling sequence of railcars, a simulation method is used to determine disassembled trains, starting and ending time of disassembling and assembling, and so forth, as shown in Figure 3.

**Figure 4:**
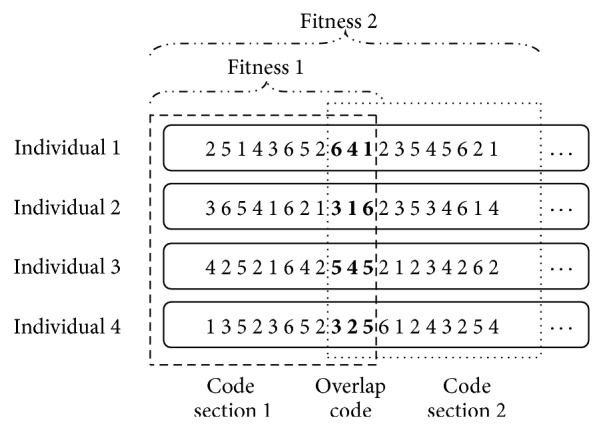
An optimization method combining subperiod rolling into GA.

With a long scale of planning horizon, it is inefficient to search the large-scale solution space of railcars' assembling sequence within the whole planning horizon. Considering that a long-term operating plan could be divided into several short period subplans, railcars' assembling sequence is optimized by combining subperiod rolling into GA in this paper. Firstly, a relative short period (e.g., 1 day) from the starting time of planning horizon is chosen, and its assembling sequence is optimized using GA. Then roll forth a new same-length period, and optimize this period's code of each individual while holding previous periods' code unchanged. The whole operating plan will be obtained by continuously optimizing each subperiod's code, as shown in Figure 4. Specially, there is an overlap code between two adjacent code sections, which contributes to their joining. Moreover, the fitness is to evaluate the quality of code sections which starts from individual subperiod's first code and ends at current period's last code. In other words, it evaluates the operating plan till the end of current period.

A more detailed explanation of the optimization method shown in Figure 4 is given as follows. The code sections 1 and 2 of four individuals in Figure 4 represent the codes of the first and second short time periods, respectively. Each gene is an integer between 1 and the combination number of *C*, which represents the index of a direction combination in *C*. For example, code section “25143652641,” which means that outbound trains will be assembled with direction combinations *c*_2_, *c*_5_, *c*_1_, *c*_4_, *c*_3_, *c*_6_, *c*_5_, *c*_2_,… successively, represents the code of individual 1 in the first short time period, and code section “64123545621” expresses the code of individual 1 in the second short time period. It is necessary to point out that there is an overlap code “641” between code section 1 and code section 2 of individual 1. The representing way of other individuals' code section is similar to these. When optimizing the operating plan, the code section 1 will be optimized firstly using GA, and then, keeping the code section 1 subtracting the overlap code unchanged, GA is used again to optimize the code section 2. Moreover, while optimizing the code section 1, fitness 1 of each individual is reckoned based on the operating plan of the first short time period, but, while optimizing the code section 2, fitness 2 of each individual is calculated based on the operating plan from the first short time period to the second short time period.

## 4. A Simulation Method of Operating Plan with Given Assembling Sequence of Railcars

### 4.1. Definitions of Events

Six events related to operating plan are defined in Table 1.

Each event's occurrence depends on the satisfaction of its relative prerequisites, so its occurrence time is the time when all relative prerequisites are satisfied. Each event's occurrence would make some facilities' state changed. According to a station's current state, events' occurrence time is determined as follows.

#### 4.1.1. Inbound Train Entering

Inbound trains are allowed to enter the marshalling yard with free tracks when they arrive at station. If there are no free tracks at their arriving time, they have to wait outside of there. Denote by *t*_*EU*_ the earliest time when at least one free arrival track exists from now on and by *u* a free arrival track. If there is more than one free arrival track at the same time, it represents any one of them. So the occurrence time of inbound train entering event is the largest of *a*_i^*^_ and *t*_*EU*_; namely,

(11) $$\begin{array}{c} t_{e}=\max\left\{a_{i^{*}},t_{EU}\right\}. \end{array}$$

After this event occurs, the time of inbound train *i* ^*^ entering arrival yard is *e*_i^*^_ = *t*_*e*_, and its storage track is *u*_i^*^_ = *u*. Meanwhile, the state of track *u* transfers from being free to being occupied; that is, *i*_*u*_ = *i* ^*^.

#### 4.1.2. Disassembling Start

Denote by *t*_*H*_ the earliest time when at least one inbound train satisfies the disassembling requirements and by *p*_*h*_ the earliest time when one disassembling engine is free. Then the calculation of disassembling starting time should consider the following two situations according to the relationship between *t*_*H*_ and *p*_*h*_.

*(1) When t*_*H*_ ≥ *p*_*h*_. In this case, disassembling starting time *t*_*h*_^*s*^ is the time when inbound train satisfies the disassembling requirement; namely,

(12) $$\begin{array}{c} t_{h}^{s}=t_{H}. \end{array}$$

Suppose train *i*_*u*_ satisfies disassembling requirements on track *u*. 

*(2) While t*_*H*_ < *p*_*h*_. In this situation, disassembling starting time *t*_*h*_^*s*^ is the time of disassembling engine being free; namely,

(13) $$\begin{array}{c} t_{h}^{s}=p_{h}. \end{array}$$

As there may be more than one inbound train which satisfies disassembling requirements until the time *p*_*h*_, denote by *Ω*_*h*_ the set of these trains. In order to assemble more railcars into the next outbound train, choose train *i*_*u*_ containing maximum railcars, whose directions belong to the next assembling combination *c* ^*^. It satisfies

(14) $$\begin{array}{c} M_{i_{u}}^{c^{*}}=\max\left\{M_{i_{u^{\prime }}}^{c^{*}}\;|\;i_{u^{\prime }}\in \Omega_{h}\right\}, \end{array}$$

where *M*_*i*_*u*__^c^*^^ is the number of railcars, whose directions belong to combination *c* ^*^, on train *i*_*u*_.

When starting disassembling train *i*_*u*_ at *h*_*i*_*u*__^*s*^ = *t*_*h*_^*s*^, the disassembling engine's state would be transferred from free to busy, and the state of track *u* would be transferred from occupied to free; that is,

(15) $$\begin{array}{c} i_{h}=i_{u},\\ p_{h}=h_{i_{u}}^{s}+\frac{\left|B_{i_{u}}\right|}{\delta }+HI,\\ i_{u}=0. \end{array}$$

#### 4.1.3. Disassembling End

The event of disassembling end only occurs after starting disassembling train. If all disassembling engines do not work now, the disassembling ending time is +*∞*; otherwise, it is the time when one disassembling engine completes humping the current train *i*_*h*_. That is,

(16) $$\begin{array}{c} t_{h}^{e}=\left\{\begin{array}{cc} h_{i_{h}}^{s}+\frac{\left|B_{i_{h}}\right|}{\delta }, & i_{h}>0,\\ +\infty , & i_{h}=0. \end{array}\right. \end{array}$$

When train *i*_*h*_ ends disassembling at *h*_*i*_*h*__^*e*^ = *t*_*h*_^*e*^, its railcars staying tracks are determined as follows. If one track has stored railcars and the car number is less than its maximum storage capacity, then the railcar would be humped into this track, or else any empty track would get this railcar. Disassembling engine's state would be transferred from busy to free, and the number of storage railcars on some marshalling tracks would increase; that is,

(17) $$\begin{array}{c} i_{h}=0,\\ B_{w}=B_{w}\cup \left\{b_{i_{h}}^{k}\;|\;w_{i_{h}}^{k}=w,\;\;k=1,2,\ldots ,\left|B_{i_{h}}\right|\right\},\;\forall w. \end{array}$$

#### 4.1.4. Assembling Start

Denote by *t*_*EV*_ the time when the departure yard has free tracks, by *π* the free assembling engine, and by *p*_*π*_ = min⁡{*p*_*π*′_} its earliest free time. The number of railcars, whose directions belong to combinations *c* ^*^, reaches the minimum size of outbound train at time *t*_*A*_. Then the starting time of assembling is

(18) $$\begin{array}{c} t_{a}^{s}=\max\left\{t_{EV},p_{\pi },t_{A}\right\}. \end{array}$$

Therefore, the next outbound train *j* ^*^ will be assembled with component railcars *B*_*j*_ by engine *π*_j^*^_ = *π* at *r*_j^*^_^s^ = *t*_*a*_^*s*^. Component railcars *B*_*j*_ of train *j* ^*^ are determined as follows. Firstly, choose one direction in combination *c* ^*^ in a given sequence. Then choose an occupied marshalling track whose storage railcars' direction is the same as the selected one. If its railcars number does not exceed the maximum size of outbound train, then add them into *B*_*j*_; otherwise, choose a part of them just reaching the maximum size.

The corresponding state changes include the free assembling engine *π* turning into busy and some marshalling tracks turning into empty or their storage railcars number decreases; that is,

(19) $$\begin{array}{c} j_{\pi }=j^{*},\\ B_{w}=\frac{B_{w}}{\left(B_{j}\cap B_{w}\right)},\;\forall w. \end{array}$$

#### 4.1.5. Assembling End

The event of assembling end should only occur after starting assembling train. If all assembling engines do not work now, the assembling ending time is +*∞*; otherwise, it is the time at which one assembling engine *π* completes assembling the current train *j*_*π*_; namely,

(20) $$\begin{array}{c} t=\min\left\{\left(r_{j_{\pi^{\prime }}}^{s}+\epsilon_{f}+\left(n_{j_{\pi^{\prime }}}-1\right)*\epsilon_{a}\right)\;|\;j_{\pi^{\prime }}>0\right\}. \end{array}$$

Therefore, the assembling ending time is

(21) $$\begin{array}{c} t_{a}^{e}=\left\{\begin{array}{cc} +\infty , & \text{if}\;\;\forall j_{\pi }=0,\\ t, & \text{else}. \end{array}\right. \end{array}$$

When train *j*_*π*_ ends assembling at *r*_*j*_*π*__^*e*^ = *t*_*a*_^*e*^, assembling engine *π* turns into free, and departure track *v* storing train *j*_*π*_ turns into occupied; namely,

(22) $$\begin{array}{c} j_{v}=j_{\pi },\\ j_{\pi }=0,\\ p_{\pi }=t_{a}^{e}+HI. \end{array}$$

#### 4.1.6. Outbound Train Departure

The technical inspections and the minimum interdeparture interval requirements should be satisfied before outbound trains depart. Denote by *t*_*I*_ the time at which the minimum interdeparture intervals meet and by *t*_*Q*_ the time at which an outbound train has completed the technical inspections.

1. If *t*_*I*_ ≤ *t*_*Q*_, then the train departure time is *t*_*d*_ = *t*_*Q*_, and suppose train *j*_*v*_ completes the technical inspection firstly.
2. If *t*_*I*_ > *t*_*Q*_, then the train departure time is *t*_*d*_ = *t*_*I*_. As there may be more than one outbound train that completed the technical inspection at that time, choose train *j*_*v*_ with the maximum number of railcars so as to make more railcars depart from station.

After train *j*_*v*_ departing from station at *d*_*j*_*v*__ = *t*_*d*_, the occupied departure track *v* turns into free; namely,

(23) $$\begin{array}{c} j_{v}=0. \end{array}$$

### 4.2. The Simulation Framework and Steps

**Figure 5:**
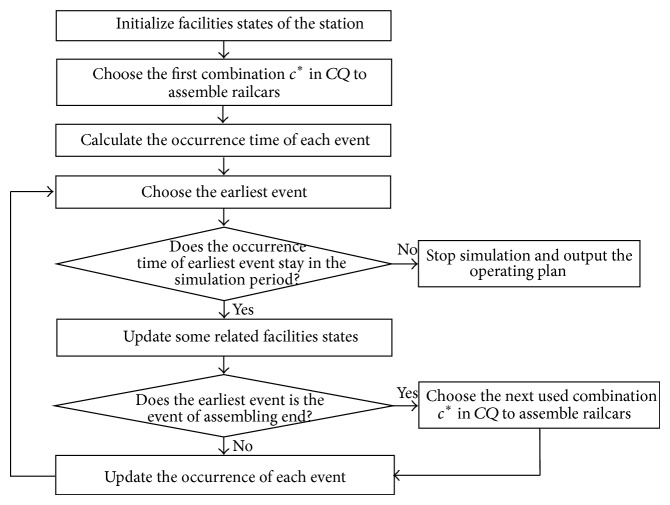
The simulation framework of operating plan.

As railcars whose directions belong to the same combination could be assembled into the same outbound train, railcars' assembling sequence is described with a sequence of direction combinations. For a given combinations sequence *CQ*, determine each event occurrence time according to facilities' usage states from the starting times of planning horizon. Then choose the earliest event, and update its relevant equipment's states. This process is repeated to obtain (1) entering time, arrival track for staying, and disassembling plans of each inbound train and (2) assembling plan, storage departure tracks, and departure times of each outbound train until the end time of the planning horizon. The simulation framework for optimizing the operating plan with a given direction combination sequence is shown in Figure 5.

Denote *S* = {{*i*_*u*_}, {*B*_*w*_}, {*j*_*v*_}, {*p*_*σ*_}, {*i*_*σ*_}, {*p*_*π*_}, {*j*_*π*_}} to describe station's states including the usage states of arrival tracks, marshalling tracks, departure tracks, disassembling engines, and assembling engines. At the start of planning horizon, the usage state of each facility is initialized as follows.

(1) All tracks of arrival, marshalling, and departure yard are empty at first; namely,
  (24) $$\begin{array}{c} i_{u}=0,\;u=1,2,\ldots ,U,\\ B_{w}=\emptyset ,\;w=1,2,\ldots ,W,\\ j_{v}=0,\;v=1,2,\ldots ,V. \end{array}$$
(2) All disassembling engines are free originally; namely,
  (25) $$\begin{array}{c} p_{\sigma }=0,\;\forall \sigma ,\\ i_{\sigma }=0,\;\forall \sigma . \end{array}$$
(3) All assembling engines are free in the beginning; namely,
  (26) $$\begin{array}{c} p_{\pi }=0,\;\forall \pi ,\\ j_{\pi }=0,\;\forall \pi . \end{array}$$

Based on the simulation framework shown in Figure 5 and events definition in Section 4.1, the simulation steps for operating plan with a given combination sequence *CQ* are designed as shown in Algorithm 1.

## 5. An Optimization Method of Long-Term Operating Plan Combining Subperiod Rolling into GA

In the following, the optimization steps of this method are presented after some key technologies of GA are being explained.

### 5.1. Individual Encoding and Decoding

**Figure 6:**
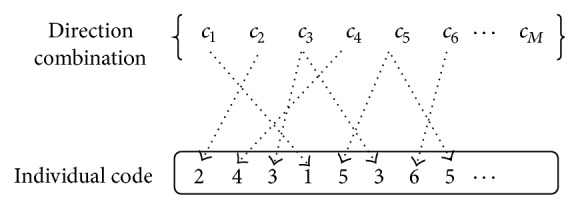
Encoding method.

Denote by |*C*| the combination number of candidate set *C*, and take the integer between 1 and |*C*| as a gene of individual code, as shown in Figure 6, where *c*_1_, *c*_2_,…, *c*_**M**_ are the direction combinations, and only railcars whose directions belong to the same combination can be assembled into the same outbound train. Thus the indexes of each combination in set *C* are 1,2,…, *M*, respectively, which will be used to form individual code. For example, the code “24315365…” represents the outbound trains assembled with direction combinations *c*_2_, *c*_4_, *c*_3_, *c*_1_, *c*_5_, *c*_3_, *c*_6_, *c*_5_,… successively. Different code positions may have the same gene value. Each gene value represents the direction combination of its number. Each code can be divided into many small sections according to the subperiods. Each section's length may differ because of the difference on assembled trains' number in each subperiod. Denote by *l*_*s*_^*g*^, *l*_*s*_^′*g*^ the starting and ending position of section *g* in individual *s*, respectively.

**Figure 7:**
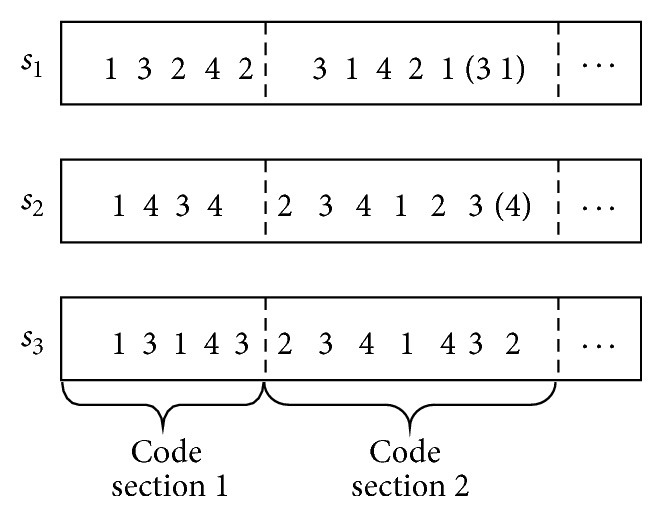
The method of keeping current code section's length to be the same.

Each cross and variation operation only handles the code section of current subperiod, and the code sections of previous subperiods would remain unchanged. As shown in Figure 7, the code section 1 of individuals *s*_1_, *s*_2_, *s*_3_ is attained after optimizing the operating plan of subperiod 1. When optimizing the operating plan of subperiod 2, only code section 2 of individuals *s*_1_, *s*_2_, *s*_3_ is optimized while code section 1 of them remains unchanged.

For the convenience of individuals cross operation, if the current subperiod's code section length of each individual varies after each genetic iteration, at this point, some new genes will be randomly generated and appended to the shorter code sections in order to keep the same length as other individual code sections. As shown in Figure 7, code section “31421” is the code section of individual *s*_1_ after previous genetic iterations. Its length is 5 while the maximum length of the code section 2 of individual *s*_3_ is 7. At the moment, two genes “31” are randomly generated and appended to the code section 2 of individual *s*_1_, which will not affect fitness's reckon of individual *s*_1_, and are simply used for keeping the same code section length as others.

### 5.2. Individual Fitness Calculation

Each individual's fitness is calculated to evaluate the code sections from code's first gene to the last gene of current subperiod's code section. Denote by *f*_*i*_(*T*_*s*_, *T*_*e*_) the fitness of individual *s*_*i*_ in subperiod [*T*_*s*_, *T*_*e*_]. Firstly, calculate railcars' staying time *Z*_*i*_ of individual *s*_*i*_ in period [0, *T*_*e*_] and transfer the optimization goal from minimizing *Z*_*i*_ to maximizing *T*_*e*_ − *Z*_*i*_; then compute fitness *f*_*i*_(*T*_*s*_, *T*_*e*_) with scale transformation as follows:

(27) $$\begin{array}{c} f_{i}\left(T_{s},T_{e}\right)=\frac{e^{\left(\left(T_{e}-Z_{i}\right)/\varphi \times \log_{0.99}x\right)}}{\sum_{s_{j}\in S}e^{\left(\left(T_{e}-Z_{j}\right)/\varphi \times \log_{0.99}x\right)}}, \end{array}$$

where *x* is the generation time, *φ* is a parameter relating to scale transformation, and its value is 150 generally.

### 5.3. Genetic Operators

The basic genetic operators of GA are selection, crossover, and mutation, which are given as follows and more details about the genetic operators can be obtained in Dimitri's book of* Omega: A Competent Genetic Algorithm for Solving Permutation and Scheduling Problems*.

#### 5.3.1. Selection Operator

New population's individuals are selected from the current population by roulette method based on their fitness. Firstly, the selection probability and cumulative probability range of each individual are reckoned according to their fitness. After that, a random number between 0 and 1 is generated, and the individual, whose cumulative probability range covers this number, is selected.

#### 5.3.2. Crossover Operator

**Figure 8:**
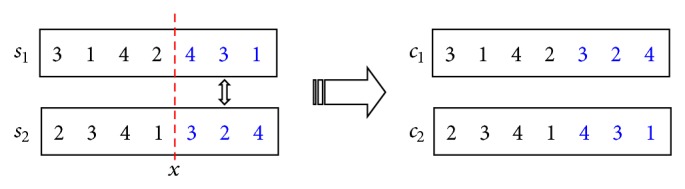
The single-point crossover method.

The single-point crossover is selected as the crossover operator here. Firstly, an intersection of the individual strings is elected randomly. Then the following part of the individual string at the intersection are exchanged to generate two new individuals. A simple example is given as shown in Figure 8. Individuals *s*_1_ and *s*_2_ are selected for crossover, the position *x* is the intersection, and individuals *c*_1_ and *c*_2_ are the new individuals after single-point crossover operator.

#### 5.3.3. Mutation Operator

The combination of uniform mutation and basic bit mutation is adopted as the mutation operator in order to search freely over the whole search space in the initial stage and only search in the local scope in the later algorithm. In other words, uniform mutation, which makes each gene value mutated with a larger probability, is adopted in the early stage, while basic bit mutation is employed in the later stage and each gene value is mutated with a smaller probability in this stage.

### 5.4. Optimization Steps of GA Combined with Subperiod Rolling

Based on the above key technologies of GA and the simulation method in Section 4, the optimization steps for optimizing the long-term operating plan are designed as shown in Algorithm 2.

## 6. Numeric Examples

In this section, based on the optimization results about operating plan of the marshalling station shown in Figure 1, (1) the convergence of the designed algorithm, (2) the effect of some algorithm parameters, and (3) the influence of arrival train flow on the algorithm will be analyzed.

The algorithm is developed with computer language C# on the platform of Microsoft Visual Studio.net and runs on the computer with the system of Microsoft Windows XP (Home Edition), RAM configuration of Pentium(R) Dual-Core CPU E5800 @ 3.20 GHZ, 3.19 GHz, 2.96 GB. For GA, its population size is *θ* = 100, its maximum and minimum crossover probability are *pc*_1_ = 0.9 and *pc*_2_ = 0.5, respectively, its maximum and minimum mutation probability are *pm*_1_ = 0.05 and *pm*_2_ = 0.005 separately, and its maximum generation count for the best individual continually keeping unchanged is *X* = 50.

Some parameters' values of arrival yard, marshalling yard, and departure yard are shown in Table 2. In addition, railcars' maximum and minimum number of arrival trains are 50 and 100, respectively, railcars directions include “AD,” “AF,” “AW,” “AY,” “AH,” “AK,” “AN,” “AP,” “AJ,” “BG,” “AR,” “AX,” and “AV,” and the arrival times of inbound trains distribute in the whole day and their fluctuation is described by their variance. According to those parameters' values, the railcars' number, direction, and arrival time of arrival trains are generated randomly. Moreover, railcars with directions belonging to the same direction combination can be assigned to the same outbound train as shown in Table 3.

### 6.1. Algorithm Convergence

**Figure 9:**
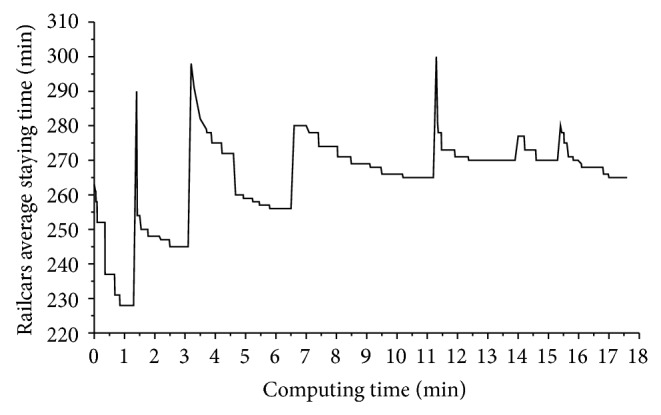
The variation relation of railcars average staying time along with the computing time.

The variation relation of railcars average staying time along with the computing time is drawn as shown in Figure 9 when optimizing the 5-day operating plan with subperiod length of 18 h and overlap length of 2 h. In this example, the average number of arrival trains per day is 30, and the variance of their arrival time is 1.

As shown in Figure 9, the 5-day planning horizon is divided into 8 subperiods, namely, [0, 1080), [960, 2040), [1920, 3000), [2880, 3960), [3840, 4920), [4800, 5880), [5760, 6840), and [6720, 7200], so the 5-day operating plan is accordingly obtained through rolling optimizing the subplans of these 8 subperiods. Their computing times are 1.3 min, 1.8 min, 3.8 min, 4.3 min, 2.7 min, 1.4 min, 1.4 min, and 0.9 min, respectively, and railcars' average staying times are 228 min, 245 min, 256 min, 265 min, 270 min, 270 min, 268 min, and 265 min per railcar. Although the computing times of each subplan are different obviously, they are acceptable. Railcars average staying times vary a little from 250 min to 270 min and from 228 min to 245 min for the 1st and 2nd subplans as all facilities are free and can disassemble and assemble trains in time in the beginning. Therefore, it is not difficult to draw the conclusion that this algorithm can converge to a satisfied plan with an acceptable computing time of 17.6 min.

### 6.2. Effect of Algorithm Parameter on the Algorithm

**Figure 10:**
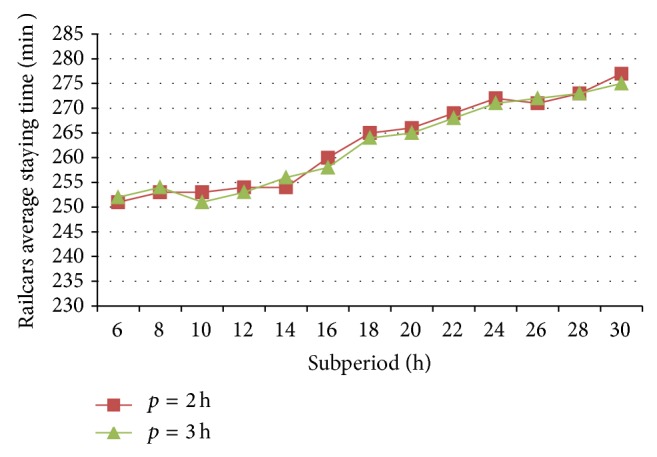
Railcars average staying time of different subperiod and overlap lengths.

**Figure 11:**
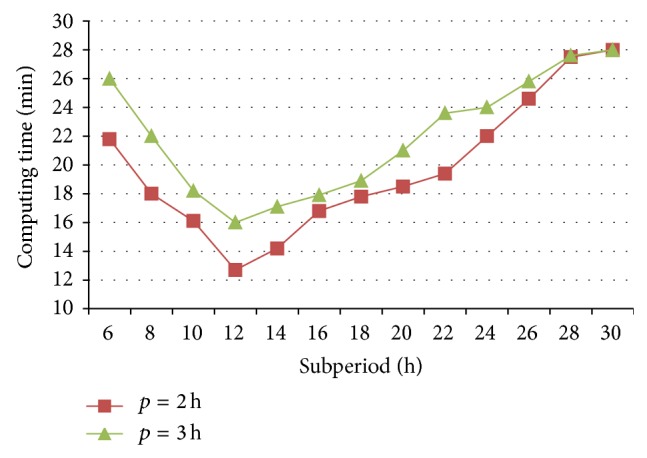
Computing times of different subperiod and overlap lengths.

When the average number of arrival trains per day is 30 and the variance of their arrival times is 1, railcars' average staying times and the computing times of 5-day operating plans are optimized with different subperiod and overlap lengths, as shown in Figures 10 and 11, respectively.

When the overlap length is 2 h, railcars average staying time stays in a narrow range of 250~255 min, but the computing time changes largely from 21.8 min to 12.7 min along with the increasing of subperiod's length from 6 h to 12 h. Then, with the continuous increase from 12 h to 30 h, not only does railcars average staying time promote faster from 253 min to 276 min, but also the computing time increases quickly from 12.7 min to 27.6 min. The same trend exists while the overlap length is 3 h. So it has a moderate subperiod length, such as 12 h in this example, with which a satisfied plan can be obtained with less computing time.

In addition, two variation curves of railcars average staying time are mainly consistent when the overlap lengths are 2 h and 3 h, respectively, but the computing time of the former is slightly less than that of the latter. Hence, it is suggested to adopt a shorter overlap to optimize the plan.

### 6.3. Influence of Inbound Train Flow on the Algorithm

The optimization results of different planning horizons and inbound train flows are shown in Table 4. In these examples, there are two planning horizons, 5 days and 10 days, three inbound trains numbers, 20 railcars, 30 railcars, and 40 railcars per day, and two variances of their arrival time, 0.5 and 1. It is found that the computing time increases obviously along with inbound trains' number increase from 20 railcars to 40 railcars per day and that railcars average staying time is optimal when inbound trains' number is 30. Besides, the arrival time variances change from 0.5 to 1 of inbound trains resulting in a slight increase in railcars' average staying time and producing a little effect on the computing time.

## 7. Conclusion

In order to provide more detailed data for analyzing the capacity utilization of marshalling station from the long-term operating plan, a model of long-term operating plan is built to minimize railcars' average staying time under the constraints of minimum time intervals, marshalling track capacities, and so forth. Its solving algorithm is designed based on GA and simulation method, in which railcars' assembling sequences are optimized by GA and then the operation plans are obtained based on the previously achieved assembling sequences through the simulation method. In order to reduce the computing time, the whole planning horizon is divided into many subperiods, which are optimized sequentially. As the optimization of each subplan converges in a short time, the whole algorithm can converge to a satisfied plan with an acceptable computing time. Moreover, an appropriate length, of subperiods helps decrease the computing time. In addition, the growing of arrival trains number leads to the fast increase in computing time, while the change of arrival time variances has a slight effect on computing time.

This paper specifically optimizes the operating plan of one type of marshalling station, in which trains of up and down directions do not interfere with each other, but in reality they may interfere at some other type of marshalling stations. Therefore, further study on optimizing the operating plans of those stations can be conducted in the future. In addition, some ignored constraints should be taken into account in the optimization of marshalling stations' operating plans in the future study, such as some railcars forbidden to disassemble through a hump.

## Figures and Tables

**Algorithm 1:**
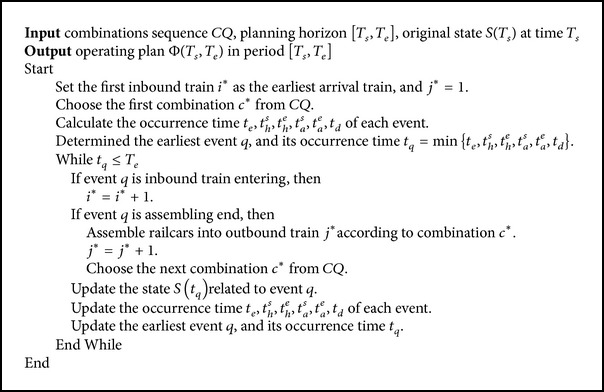


**Algorithm 2:**
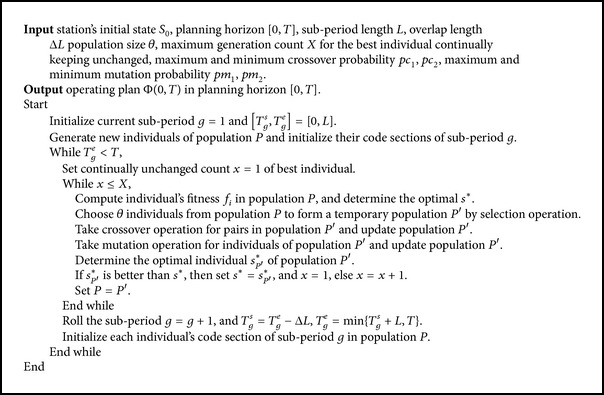


**Table 1:** Events' definition and occurrence prerequisite.

Number	Event name	Definition	Occurrence prerequisite
1	Inbound train entering	Inbound trains enter arrival yard	① Inbound train arrives at station ② Arrival yard has free tracks

2	Disassembling start	Disassembling engine pushes inbound train up the hump for disassembling	① Disassembling engine is free. ② There exist trains in the arrival yard with technical inspections completed ③ Marshalling yard has enough tracks to store disassembled railcars

3	Disassembling end	All railcars of current disassembled train have run on assigned marshalling tracks	① Disassembling train is completed

4	Assembling start	Assembling engine starts pulling railcars from marshalling track to assemble outbound train	① Assembling engine is free ② Marshalling yard has enough railcars which can be assembled into the same outbound train ③ There exist free tracks in the departure yard

5	Assembling end	Assembling engine stops pulling railcars and an outbound train is formed	① Assembling train is completed

6	Outbound train departure	Outbound train departs from departure yard	① Departure yard has outbound trains having technical inspections finished ② Minimum interdeparture interval is satisfied

**Table 2:** Some parameters' values of marshalling station.

Number	Parameter name	Parameter value
1	*U*	10
2	*W*	42
3	*V*	7
4	*ξ*	60
5	|*σ*|	1
6	|*π*|	2
7	*DI*	10 min
8	*δ*	3 railcars per min
9	*HI*	10 min
10	*AI*	5 min
11	*τ*	45 min
12	*ϵ*_*f*_	10 min
13	*ϵ*_*a*_	15 min per pull
14	Γ_min⁡_	50 railcars
15	Γ_max⁡_	140 railcars

**Table 3:** Directions combination for assembling outbound train.

Number	Combination
1	AD, AF
2	AF, AW, and AY
3	AV
4	AH, AK
5	AR, AW, and AY
6	AX
7	AN, AP, AJ, and BG

**Table 4:** The optimization results of different planning horizons and inbound train flows.

Planning horizon (d)	Inbound trains number	Variance of arrival time	Railcars average staying time (min)			Computing time (min)		
			*L* = 6 h	*L* = 12 h	*L* = 24 h	*L* = 6 h	*L* = 12 h	*L* = 24 h
5	100	0.5	268	267	271	9.5	**7.2**	**9.9**
		1	271	272	279	9.7	7.3	9.6
	150	0.5	246	247	256	22.1	13.4	22.7
		1	251	254	273	21.8	12.7	22.1
	200	0.5	317	318	339	47.2	30.9	44.7
		1	319	321	344	48.4	31.6	43.5

10	200	0.5	270	273	282	20.8	19.2	21.2
		1	274	276	283	21.3	18.2	20.5
	300	0.5	248	251	257	68.2	46.7	56.7
		1	253	258	262	67.5	47.2	57.5
	400	0.5	324	325	332	110.2	85.9	96.8
		1	324	326	334	110.4	86.3	97.2

## Notations

### Parameters

U: Track number of train arrival yard
u: u=1,2,…,U arrival track
W: Track number of railcar marshalling yard
w: w=1,2,…,W marshalling track
ξ: The maximum railcars number that can be held in each marshalling track
V: Track number of train departure yard
v: v=1,2,…,V departure track
N: Inbound train number within planning horizon
$\left|\sigma \right|\text{:}$: Disassembling engine’s count
σ: $\sigma =1,2,\ldots ,\left|\sigma \right|$ disassembling engine
$\left|\pi \right|\text{:}$: Assembling engine’s count
π: $\pi =1,2,\ldots ,\left|\pi \right|$ assembling engine
c: Railcar direction combination: railcars whose directions belong to the same combination could be assembled into the same outbound train
C: C={c} set of railcar direction combinations
i: i=1,2,…,N inbound train
$a_{i}\text{:}$: Arrival time of inbound train i at station
$B_{i}\text{:}$: Railcar sequence of train i
$\left|B_{i}\right|\text{:}$: Railcar number of train i
$b_{i}^{k}\text{:}$: kth railcar of train i
$d_{i}^{k}\text{:}$: kth railcar’s direction of train i
$i_{b}\text{:}$: Inbound train of railcar b
M: Outbound train number within planning horizon
j: j=1,2,…,M outbound train
DI: Minimum train interdeparture time
HI: Minimum disassembling interval
AI: Minimum assembling interval
δ: Humping rate, namely, the number of disassembling railcars per minute
$\epsilon_{f}\text{:}$: Average time to perform first track pull
$\epsilon_{a}\text{:}$: Average time to perform an additional track pull
$\Gamma_{\min}\text{:}$: Minimum railcars number of outbound train
$\Gamma_{\max}\text{:}$: Maximum railcars number of outbound train
τ: Technical inspection time for both inbound and outbound trains
$\left[0,T\right]\text{:}$: Planning horizon.

### Variables

$e_{i}\text{:}$: Arrival time of train i at arrival yard; if no free arrival tracks exist when train i arrives at station, $e_{i}\neq a_{i}$; otherwise, $e_{i}=a_{i}$
$u_{i}\text{:}$: Arrival track occupied by train i
$\sigma_{i}\text{:}$: Disassembling engine working for train i
$h_{i}^{s}\text{:}$: Starting time of disassembling train i
$h_{i}^{e}\text{:}$: Ending time of disassembling train i
$w_{i}^{k}\text{:}$: Assigned marshalling track for kth railcar after humping train i
$c_{j}\text{:}$: Direction combination for assembling train j
$\pi_{j}\text{:}$: Assembling engine working for train j
$r_{j}^{s}\text{:}$: Starting time of assembling train j
$r_{j}^{e}\text{:}$: Ending time of assembling train j
$B_{j}\text{:}$: Railcar sequence of train j
$v_{j}\text{:}$: Departure track occupied by train j
$d_{j}$: Departure time of train j.

### Intermediate Variables

$\Phi (T_{s},T_{e})\text{:}$: Operating plan in period $\left(T_{s},T_{e}\right)$
$i_{u}\text{:}$: Inbound train stored in arrival track u now; if $i_{u}=0$, it means no train occupies this track at this time
$B_{w}\text{:}$: Railcars set stored in marshalling track w now
$\left|B_{w}\right|\text{:}$: Number of railcars stored in marshalling track w now
$j_{v}\text{:}$: Outbound train stored in departure track v now; if $j_{v}=0$, it shows that no train occupies this track at this time
$p_{\sigma }\text{:}$: The earliest time when the disassembling engine σ can work from now on
$p_{\pi }\text{:}$: The earliest time when the assembling engine π can work from now on
$i^{*}\text{:}$: The next inbound train which will arrive at station
$j^{*}\text{:}$: The number of trains which has been assembled or is being processed now
$c^{*}\text{:}$: Direction combination chosen for assembling next outbound train
$i_{\sigma }\text{:}$: Train for which disassembling engine σ works now; if $i_{\sigma }=0$, it shows the engine σ is free
$j_{\pi }\text{:}$: Train for which assembling engine π works now; if $j_{\pi }=0$, it shows engine π is free.
